# Mechanical Dentistry

**Published:** 1874-06

**Authors:** WM. O. Kulp

**Affiliations:** Davenport, Iowa.


### ARTICLE III.

### *Mechanical Dentistry.*

BY WM. O. KULP, DAVENPORT, IOWA.

Read before the Illinois State Dental Society.

This old term, "mechanical dentistry," has in it a tingle of odium to many good men in our profession. I can't blame them for it, for are we not a step in advance to a mechanical trade, and does not the term smack of a trade rather than a department of an educated, scientific profession, as ours claims to be? We, therefore, want those in our societies to feel that they have a much weightier responsibility than they would have were they members of a

"trade union." We would not willingly hear surgeons called "mechanical doctors," or surgical appliances "mechanical medicines," yet it would seem just as elegant as to say the "mechanical dentist," or "mechanical dentistry." The term is not inappropriate in either case, for there is much mechanical skill required in the performance of the various operations in each specialty; still it does not sound professional. I, therefore, propose to christen the department of our profession anew, and call it *artistic dentistry*, instead of mechanical dentistry, believing the term to be more appropriate and professional, and it, therefore, will be more acceptable to the profession. It may give the specialty a new standing in the eyes of professional men, and men employed in it may be stimulated to greater efforts to achieve for their department that higher position of perfection which we all so much long to see it occupy.

The artistic dentist should be by nature endowed with much inventive and imitative ingenuity and patient perseverance. He should be a judge of colors, light and shade, form and size, and should have the acquired knowledge of anatomy, physiology, chemistry, natural philosophy, and enough of surgery and general medicine to be able to treat successfully diseased conditions of the mouth, and extract teeth. Let this be the standard, and our profession will not be borne down by its odious members, and humanity will derive the full benefits of this merciful calling. Then the day for impostors and impositions emanating from our unworthy adherents will cease. There perhaps never was a time in the history of our profession when this department was more in a tumult than it is to-day—beset on all sides with patents, patent medicines, patent instruments, patent appliances, patent bases, etc.—some good, some indifferent, others wholly worthless—humbugs. It would take a fortune of money, and all the time at our command, to "keep the run of," and try all the new things that clamor for admission into our patients' mouths and into our laboratories. A few of the most worthy I will consider, aiming only to open the discussion upon this whole subject.

What are the requirements of the substance upon which to mount artificial teeth? I answer briefly:

1st. It should be a substance wholly harmless, therapeutically or chemically, to the wearer.

2nd. It should be impervious to the secretions of the mouth, either in a vitiated or normal condition; also to all chemical agents that are likely to be taken into the mouth, either as food, luxury or medicines.

3rd. It should possess sufficient strength, with the least possible thickness, and at the same time should be malleable or plastic enough to admit of being used in building out the absorbed alveoli to any desired thickness, without becoming too heavy and bunglesome to be worn by the patient with comfort.

4th. It should be the natural color of the gums and vermillion border of the lips; this should be capable of being deepened or lessened, as each case may require, as no two persons have precisely the same colored gums.

5th. It should be cheap, so that the poor can avail themselves of the comforts and necessity of artificial dentures.

This leads us to the inquiry, what have we in the profession to-day that "fills the bill?" Echo answers, what? Is gold plate the substance? It has the first two requirements, but there it ends. In combination with rubber, it has another, and I believe is most entitled to the palm of any material in the profession to-day—except cheapness, when rubber alone is the best of all cheap bases, while its rivals—gutta percha, coralline, piroxyline—are attracting much attention, mainly because of the dark and lowering cloud overhanging the rubber question. They are not equal to the race thus far, for their objections are much more numerous, while their advantages are not worthy of mention. So far as beauty and strength are concerned, Dr. Allen's continuous gum work excels all other substances in the profession. But whoever succeeds in overcoming the few serious objections to the porcelain base, in limited use, will set his name by the side of an Arthur, Atkinson and a Barnum, for we have nothing that approximates so nearly to the *ne plus ultra* as does this substance. Who will be the genius? To whom shall we render this honor? Nations will rise up and call the successful one blessed. I do not mention silver and the metal compounds offered to the profession in these days, for I do not consider them fit to be put into our patients' mouths.

---

### SELECTED ARTICLES.

